# Supplementary material for: End Sequence Analysis Toolkit (ESAT) expands the extractable information from single-cell RNA-seq data
Source: Genome Res. 2016 Oct;26(10):1397–410. doi: 10.1101/gr.207902.116 (PMC5052061; doi:10.1101/gr.207902.116)
Supplement: Supplemental Material [file supp_gr.207902.116_Supp_Materials.tar.gz › ESAT-esat_v0.1/doc/html/classbroad_1_1core_1_1util_1_1_c_l_util.html]

ESAT: broad.core.util.CLUtil Class Reference


ESAT


- Main Page
- Related Pages
- Packages
- Classes
- Files

- Class List
- Class Index
- Class Hierarchy
- Class Members

All Classes Namespaces Files Functions Variables Enumerator Pages

- broad
- core
- util
- CLUtil

Classes |
Public Member Functions |
Static Public Member Functions |
List of all members

broad.core.util.CLUtil Class Reference

|  |  |
| --- | --- |
| Classes | |
| class | **ArgumentMap** |
|  | |

|  |  |
| --- | --- |
| Public Member Functions | |
|  | CLUtil () |
|  | |

|  |  |
| --- | --- |
| Static Public Member Functions | |
| static ArgumentMap | getParameters (String[] args, String usage, String defaultTask) |
|  | |
| static ArgumentMap | getParameters (String[] args, String usage) |
|  | |
| static void | writeLeftJustifiedField (BufferedWriter bw, String value, int fieldSize) throws IOException |
|  | |
| static void | writeRightJustifiedField (BufferedWriter bw, String value, int fieldSize) throws IOException |
|  | |
| static String | replaceFileExtension (String fileName, String newExtension) |
|  | |
| static double | log2 (double x) |
|  | |
| static< T > List< T > | listFromArray (T[] array) |
|  | |

## Constructor & Destructor Documentation

|  |  |  |  |  |
| --- | --- | --- | --- | --- |
| broad.core.util.CLUtil.CLUtil | ( |  | ) |  |

## Member Function Documentation

|  |  |  |  |
| --- | --- | --- | --- |
| static ArgumentMap broad.core.util.CLUtil.getParameters | ( | String[] | *args*, |
|  |  | String | *usage*, |
|  |  | String | *defaultTask* |
|  | ) |  |  |

static

Here is the caller graph for this function:

|  |  |  |  |
| --- | --- | --- | --- |
| static ArgumentMap broad.core.util.CLUtil.getParameters | ( | String[] | *args*, |
|  |  | String | *usage* |
|  | ) |  |  |

static

Here is the call graph for this function:

|  |  |  |  |  |  |
| --- | --- | --- | --- | --- | --- |
| static <T> List<T> broad.core.util.CLUtil.listFromArray | ( | T[] | *array* | ) |  |

static

|  |  |  |  |  |  |
| --- | --- | --- | --- | --- | --- |
| static double broad.core.util.CLUtil.log2 | ( | double | *x* | ) |  |

static

|  |  |  |  |
| --- | --- | --- | --- |
| static String broad.core.util.CLUtil.replaceFileExtension | ( | String | *fileName*, |
|  |  | String | *newExtension* |
|  | ) |  |  |

static

|  |  |  |  |
| --- | --- | --- | --- |
| static void broad.core.util.CLUtil.writeLeftJustifiedField | ( | BufferedWriter | *bw*, |
|  |  | String | *value*, |
|  |  | int | *fieldSize* |
|  | ) |  | throws IOException |

static

|  |  |  |  |
| --- | --- | --- | --- |
| static void broad.core.util.CLUtil.writeRightJustifiedField | ( | BufferedWriter | *bw*, |
|  |  | String | *value*, |
|  |  | int | *fieldSize* |
|  | ) |  | throws IOException |

static

---

The documentation for this class was generated from the following file:

- java/broad/core/util/CLUtil.java


---

Generated on Fri Aug 15 2014 15:10:57 for ESAT by  

 1.8.7
